# Neural hyperactivity in the amygdala induced by chronic treatment of rats with analgesics may elucidate the mechanisms underlying psychiatric comorbidities associated with medication-overuse headache

**DOI:** 10.1186/s12868-016-0326-z

**Published:** 2017-01-03

**Authors:** Aree Wanasuntronwong, Ukkrit Jansri, Anan Srikiatkhachorn

**Affiliations:** 1 Department of Oral Biology, Faculty of Dentistry, Mahidol University, 6 Yothi Road, Ratchathewi, Bangkok, 10400 Thailand; 2 Department of Physiology, Faculty of Medicine, Chulalongkorn University, 1874 Rama 4 Road, Pathumwan, Bangkok, 10330 Thailand; 3 International Medical College, King Mongkut’s Institute of Technology, 1 Chalongkrung Road, Lad Krabang, Bangkok, 10520 Thailand

**Keywords:** Acetaminophen, Amygdala, Anxiety, Aspirin, Medication-overuse headache, Migraine

## Abstract

**Background:**

Patients with medication-overuse headache suffer not only from chronic headache, but often from psychiatric comorbidities, such as anxiety and depression. The mechanisms underlying these comorbidities are unclear, but the amygdala is likely to be involved in their pathogenesis. To investigate the mechanisms underlying the comorbidities we used elevated plus maze and open field tests to assess anxiety-like behavior in rats chronically treated with analgesics. We measured the electrical properties of neurons in the amygdala, and examined the cortical spreading depression (CSD)-evoked expression of Fos in the trigeminal nucleus caudalis (TNC) and amygdala of rats chronically treated with analgesics. CSD, an analog of aura, evokes Fos expression in the TNC of rodents suggesting trigeminal nociception, considered to be a model of migraine.

**Results:**

Increased anxiety-like behavior was seen both in elevated plus maze and open field tests in a model of medication overuse produced in male rats by chronic treatment with aspirin or acetaminophen. The time spent in the open arms of the maze by aspirin- or acetaminophen-treated rats (53 ± 36.1 and 37 ± 29.5 s, respectively) was significantly shorter than that spent by saline-treated vehicle control rats (138 ± 22.6 s, *P* < 0.001). Chronic treatment with the analgesics increased the excitability of neurons in the central nucleus of the amygdala as indicated by their more negative threshold for action potential generation (–54.6 ± 5.01 mV for aspirin-treated, –55.2 ± 0.97 mV for acetaminophen-treated, and –31.50 ± 5.34 mV for saline-treated rats, *P* < 0.001). Chronic treatment with analgesics increased the CSD-evoked expression of Fos in the TNC and amygdala [18 ± 10.2 Fos-immunoreactive (IR) neurons per slide in the amygdala of rats treated with aspirin, 11 ± 5.4 IR neurons per slide in rats treated with acetaminophen, and 4 ± 3.7 IR neurons per slide in saline-treated control rats, *P* < 0.001].

**Conclusions:**

Chronic treatment with analgesics can increase the excitability of neurons in the amygdala, which could underlie the anxiety seen in patients with medication-overuse headache.

**Electronic supplementary material:**

The online version of this article (doi:10.1186/s12868-016-0326-z) contains supplementary material, which is available to authorized users.

## Background

Medication-overuse headache (MOH) is defined by the International Headache Society as chronic headache that occurs 15 or more days a month induced as a consequence of medication for acute or symptomatic headache for more than 3 months. This clinical syndrome is common. Population-based studies found the prevalence of MOH to be from 0.7 to 1.7%, with higher preponderance in women (74%) than in men (26%) [1]. MOH is believed to be an interaction between the medication used excessively and susceptible patients, mainly those with migraine or tension-type headache (or both). A study in an interdisciplinary pain clinic showed that the odds ratio for a patient with medication overuse to have chronic headache if they had a history of primary headache, compared with patients without a primary headache syndrome, was 13.1 [2].

Patients with MOH suffer not only from chronic headache, but often also from comorbid psychiatric symptoms. Compared with patients who have episodic migraine and controls without headache, patients with MOH showed a greater risk of depressive, obsessive–compulsive, generalized anxiety, and panic disorders [3, 4]. Patients with MOH also tended to be more susceptible to dependency on the overused medications, resembling substance abuse [4]. The cause-and-effect relationship between chronic headache and these psychiatric comorbidities is controversial. These psychiatric manifestations may be viewed as a consequence of chronic pain, because coexisting depression and anxiety are common in pain syndromes other than headache. Alternatively, the psychiatric comorbidities and headache may be the result of a common trigger, being chronic exposure to the acute medication.

Previously, our group has shown that chronic treatment with analgesics can increase cortical excitability and facilitate trigeminal nociception in animal models of headache [5, 6]. We also showed that these changes resembled those observed in rats with decreased serotonin (5-hydroxytrptamine; 5-HT) levels [7, 8]. Relatively low levels of 5-HT have been found in the platelets of patients with MOH compared with controls without headache [9]. It is therefore possible that cortical hyperexcitability and nociceptive facilitation may be the result of a derangement of the endogenous 5-HT-dependent pain control system. An alteration in this control system could also alter the function of subcortical structures governing the affective system.

The present study was conducted to investigate whether chronic treatment with analgesics can alter subcortical structures involved in the control of anxiety. We compared the effect of chronic treatment with aspirin and acetaminophen, analgesics commonly used by patients with chronic headache. To avoid the known effect of sex hormone interaction with chronic analgesic drug treatment, we used a model of medication-overuse produced in male rats. To investigate the mechanisms underlying the comorbidity of anxiety associated with medication overuse we assessed anxiety-like behavior in chronically treated rats using elevated plus maze and open field tests. We compared the electrical properties of neurons in central nucleus of the right amygdala, a structure that may play important role in the pathogenesis of depression and anxiety, in rats chronically treated with analgesics, and saline-treated vehicle control rats. To investigate the mechanisms underlying headache and the psychiatric comorbidities, we examined the expression of Fos in the trigeminal nucleus caudalis (TNC) at level of C1 and C2 in the spinal cord level and in the amygdala evoked by cortical spreading depression (CSD), an analog of the aura that precedes migraine.

## Methods

### Animals

Adult male Wistar rats weighing 200–250 g were purchased from the National Laboratory Animal Center, Mahidol University, Nakhon Pathom, Thailand. Rats were housed in stainless-steel cages in a ventilated room under a 12-h dark–light cycle, and were allowed free access to food and water. All of the protocols used in this study were approved by the Animal Care and Use Committee, Faculty of Medicine, Chulalongkorn University, Bangkok, Thailand (CU-ACUC No. 16/57).

### Drugs and treatments

Aspirin (A2093) was purchased from Sigma-Aldrich. Aspirin does not usually dissolve fully in normal saline. We carefully added NaH_2_CO_3_ to an aspirin-in-saline solution, while monitoring the pH until it reached 7.0, and presented as a clear solution. The aspirin solution was freshly prepared before use. The dose of aspirin used in the present experiments was 100 mg/kg body weight according to its antinociceptive effect [10]. Acetaminophen (paracetamol) was purchased from T.P. Drug Laboratories (Bangkok, Thailand). Each ampoule contained 300 mg/2 mL. The dose of acetaminophen used in the present experiments was 200 mg/kg [6–8, 11]. Normal saline was administered to rats in the vehicle control group. The volume of all drug injections was calculated according to standard criteria (intraperitoneally 10 mL/kg) [12]. Doses of analgesics were chosen based on the presence of efficacy without serious adverse effects [6–8, 10, 11]. Intraperitoneal injections of the calculated dose or vehicle control were made daily from 8:00 to 9:00 am.

### Study design

The present study included two experiments. The first experiment aimed to determine the effect of chronic analgesic treatment on anxiety-like behavior and the electrical activity of neurons in the amygdala. Using computer-generated random table, rats were divided into three groups (10 rats each) receiving aspirin, acetaminophen, or normal saline. The number of rats in each group was guided by our previous study [6–8]. Aspirin (100 mg/kg), acetaminophen (200 mg/kg), or normal saline vehicle (10 mL/kg) was administered intraperitoneally once daily for 30 days. Twenty-four hours after the final dose, anxiety-like behavior was measured using elevated plus maze and open field tests. After measuring behavior, the rats were humanely killed, their brains removed, and slices prepared for electrophysiological measurement of neurons in the amygdala (two brain slices per rat).

The second experiment aimed to investigate the effect of chronic analgesics treatment on activation of amygdala neurons evoked by CSD that induced trigeminal nociception. In this experiment, studied analgesics were administered as they were in the first experiment (10 rats per group). Twenty-four hours after the final dose, rats were prepared for activation of CSD using crystalline granules of KCl (3 mg). Cortical activity was monitored for 1 h. After completion, rat brains were removed and prepared for Fos immunohistology. We compared the expression of Fos in the TNC and amygdala of rats from the three groups.

To exclude the influence of hepatotoxicity possibly induced by medication, the rat livers were removed for histopathological examination. Hepatotoxicity indicators used in present study were presence of centrilobular or panacinar necrosis and sinusoidal congestion. All observations were made by investigators who were blinded to the rat treatments.

## Experiment 1

The effect of chronic treatment with analgesics on anxiety-like behavior and electrical activity of neurons in the amygdala.

### Measurement of anxiety-like behavior—elevated plus maze test

Twenty-four hours after the final dose of drug administration, anxiety-like behavior was measured using an elevated plus maze according to a standard method described by Pellow et al. [13]. Briefly, an elevated plus maze consisted of four arms connected by a central square (10 cm × 10 cm). Two of which were open arms (50 cm × 10 cm) and the other two were enclosed arms (50 cm × 10 cm × 40 cm) with same type of arm opposite to each other. The apparatus was elevated 50 cm above the floor. The arms were connected by a central 10 cm × 10 cm^2^. Each rat was placed in the center square of the maze facing an open arm and allowed to explore freely for 5 min. The rat was considered to have entered an arm when all 4 limbs were inside the arm. 70% ethanol solution was used to clean the apparatus after each trial. (1) Time spent in open arms; (2) number of open arm entries; (3) time spent in closed arms; (4) number of closed arm entries; (5) time spent on the center platform; and (6) number of center platform crossings are recorded during the 5 min test period. All behavior variables were scored by an investigator who was blinded to the rat treatments.

### Measurement of anxiety-like behavior—open field test

Black plastic box (76 cm long × 57 cm wide × 50 cm high) with a 48-square grid floor (8 × 6 squares, 9.5 cm per side) was used in open field test (as modified from Rex et al. [14]). After the elevated plus maze test session, the open field test was conducted by placing the rat in center of the open field. The field arena was divided into 3 parts (outer, 8 × 6 squares next to the wall; middle, 6 × 4 squares next to the outer area; and inner, 4 × 2 squares in the center area). The rat was allowed to explore the arena for 5 min. Time spent in each part represented the anxiety-like behavior. The number of total crosses the rat made during 5 min session was recorded as its locomotor activity. The experiments were recorded by a digital video camera recorder for later analysis. After each trial, the plate was cleaned with a 70% ethanol solution. All behavioral variables were scored by an investigator who was blinded to the rat treatments.

### Preparation of the amygdala slices

One day after behavioral measurements, rats were decapitated under isoflurane anesthesia (Fisher Scientific, Hanover Park, IL). Their brains were harvested and submerged in ice cold modified Ringer’s solution containing 234 mM sucrose, 2.5 mM KCl, 1.25 mM NaH_2_PO_4_, 10 mM MgSO_4_, 0.5 mM CaCl_2_, 26 mM NaHCO_3_, and 11 mM glucose, and was sparged with 95% O_2_/5% CO_2_, pH 7.4 for 2–3 min. Coronal slices (about 300 μm thick) of the right amygdala (approximately –1.46 mm to bregma) were cut using a microtome (Vibratome 1500, Vibratome, Bannockburn, IL) and incubated in standard Ringer’s solution (125 mM NaCl, 2.5 mM KCl, 2 mM CaCl_2_, 1 mM MgCl_2_, 26 mM NaHCO_3_, 1.25 mM NaH_2_PO_4_, and 25 mM Glucose sparged with 95% O_2_/5% CO_2_, pH 7.4) at room temperature for 1 h before recording. After incubation, recorded slices were placed individually in a recording chamber on the stage of an upright microscope (BX51W1; Olympus, Tokyo, Japan) and were continuously superfused with standard Ringer’s solution with flow rate of 3–5 mL/min using a peristaltic pump (Minipuls 3, Gilson, Villiers, France).

### Brain slice neuronal recording

Whole-cell patch-clamp recordings were made of neurons in slices of the lateral subdivision of the central nucleus of the right amygdala (CeL) under visual control using patch pipettes connected to a patch-clamp amplifier (Axopatch 200B, Axon Instruments, Foster City, CA). The patch pipettes were pulled from borosilicate glass capillaries (B150-86-10, 1.5 mm outside diameter, Sutter Instruments, Novato, CA) using a horizontal electrode puller (P-97; Sutter Instruments). The pipettes were filled with a solution containing: 140 mM K-gluconate, 20 mM KCl, 0.2 mM ethylene glycol tetraacetic acid, 2 mM MgCl_2_, 2 mM Na_2_ATP, 0.5 mM Na_3_GTP, 10 mM HEPES, and 0.1 mM spermine (pH 7.4). –10 mV liquid junction potential between the Ringer’s solution and the gluconate-based intrapipette solution was estimated. Therefore, the actual membrane potential was corrected by this value. The intrapipette solution was 280–290 mOsm/L. The electrodes resistance was 5–8 MΩ in bath solutions. All recordings were performed at 32–34 °C. One to three neurons were recorded per slice. Data were acquired through a low-pass filter at 3 kHz (8-pole Bessel filter) at a sampling rate of 10 kHz. Stimulus generation and data acquisition were performed using a pClamp 10.2 operated via a Digidata 1440A interface board (Axon Instruments) on a personal computer. Data were analyzed later using Clampfit software (Axon Instruments).

## Experiment 2

The effect of chronic treatment with analgesics on activation of amygdala neurons evoked by CSD that induced trigeminal nociception.

### Extracellular cortical activity recording and CSD induction

One day after the final dose of analgesic administration, rats were anesthetized with an intraperitoneal injection of sodium pentobarbital (60 mg/kg). Their heads were fixed to the head holder of a stereotaxic frame. For direct current (DC) recordings, a small window on the skull (2 mm in diameter) was created in the right frontal bone (3 mm anterior to the bregma and 2 mm lateral to the midline). Dura mater was then carefully removed and a glass microelectrode for detecting negative DC potential was inserted into the frontal cortex using a hydraulic micromanipulator (Narishige, Tokyo, Japan) to a depth of 500 μm. An Ag/AgCl reference electrode was placed on the skin on the back of the rat. A parietal window (7 mm posterior and 3 mm lateral to the bregma) was prepared to elicit CSD by application of crystalline granules of KCl (3 mg) onto intact dura mater overlying parietal cortex. The granules were washed away with synthetic interstitial fluid at the end of the CSD wave at approximately 1 h (method modified from Leão [15]). The DC electrical signal was amplified using a microelectrode amplifier (Nihon Kohden, Tokyo, Japan). An MP100 data acquisition system (Biopac Systems, Goleta, CA) was used to convert analog data into digital format which were then analyzed using AcqKnowledge acquisition software (Biopac Systems). The number of CSD waves occurred within 60 min was determined.

### Fos immunohistochemistry

Two hours after KCl was applied onto the intact dura mater overlying the parietal cortex, rats were deeply anesthetized with sodium pentobarbital overdose. After that they were transcardially perfused with 0.1 M phosphate buffer pH 7.4 (PBS), followed by 4% paraformaldehyde in 0.1 M PBS, pH 7.4. The brain and spinal cord were cut into a block containing C1 and C2 of the spinal cord, and amygdala areas. After immersion in 30% sucrose until saturated for cryoprotection, the block was sectioned coronally on a cryostat and 30 μm thick slices were collected into cold PBS. Serial sections were collected from brains of rats from the three groups for Fos immunohistology using a free-floating technique. After pretreatment with 1% H_2_O_2_ in PBS for 30 min for endogenous peroxidase activity quenching and 3% normal goat serum in PBS for 1 h for nonspecific binding sites blocking, sections were then incubated with rabbit anti-Fos polyclonal antibody (sc-52 lot No. A-1915; Santa Cruz Biotechnology, Dallas, TX) at 1:1000 for 24 h at 4 °C. After incubation, biotinylated secondary goat anti-rabbit IgG (BA-1000; Vector Laboratories, CA, USA) was applied to sections to detect the primary. The sections were then incubated with avidin DH: biotinylated horseradish peroxidase H complex (ABC kit; Vector Laboratories, CA). The Fos immunoreactivity was visualized using peroxidase reaction by incubating the sections in 0.03% 3,3′-diaminobenzidine tetrahydrochloride and 0.008% H_2_O_2_ in 0.05 M Tris–HCl for 8–10 min. Finally, the sections were mounted on glass slides, dehydrated in ethanol, cleared in xylene, air dried, and coverslipped. Five Fos-stained sections per C1–C2 or amygdala level per rat were chosen for counting cells. Fos-immunoreactive (IR) cells in Rexed laminae I and II were counted and expressed as number of cells per section. The cell counting was conducted by an investigator who was blinded to rat treatments.

### Statistical analyses

Data were analyzed using PASW Statistics for Windows (version 18; SPSS Inc, Chicago, IL). All variables are expressed as mean ± SD. Differences between the various groups were tested using a one-way analysis of variance (ANOVA) followed by a Dunnett test. All analyses used a two-tailed hypothesis testing method. *P* < 0.05 was considered to be significant.

## Results

Chronic aspirin and acetaminophen treatment did not alter general rat behavior, including feeding. The average body weights of rats on the day of behavioral testing were comparable between the groups (317 ± 14.7 g for aspirin-treated rats, 309 ± 7.5 g for acetaminophen-treated rats, and 316 ± 5.4 g for saline-treated control rats). Liver toxicity was excluded by histology, which demonstrated no hepatocellular necrosis.

### Effect of chronic treatment with analgesics on anxiety-like behavior

**Fig. 1:**
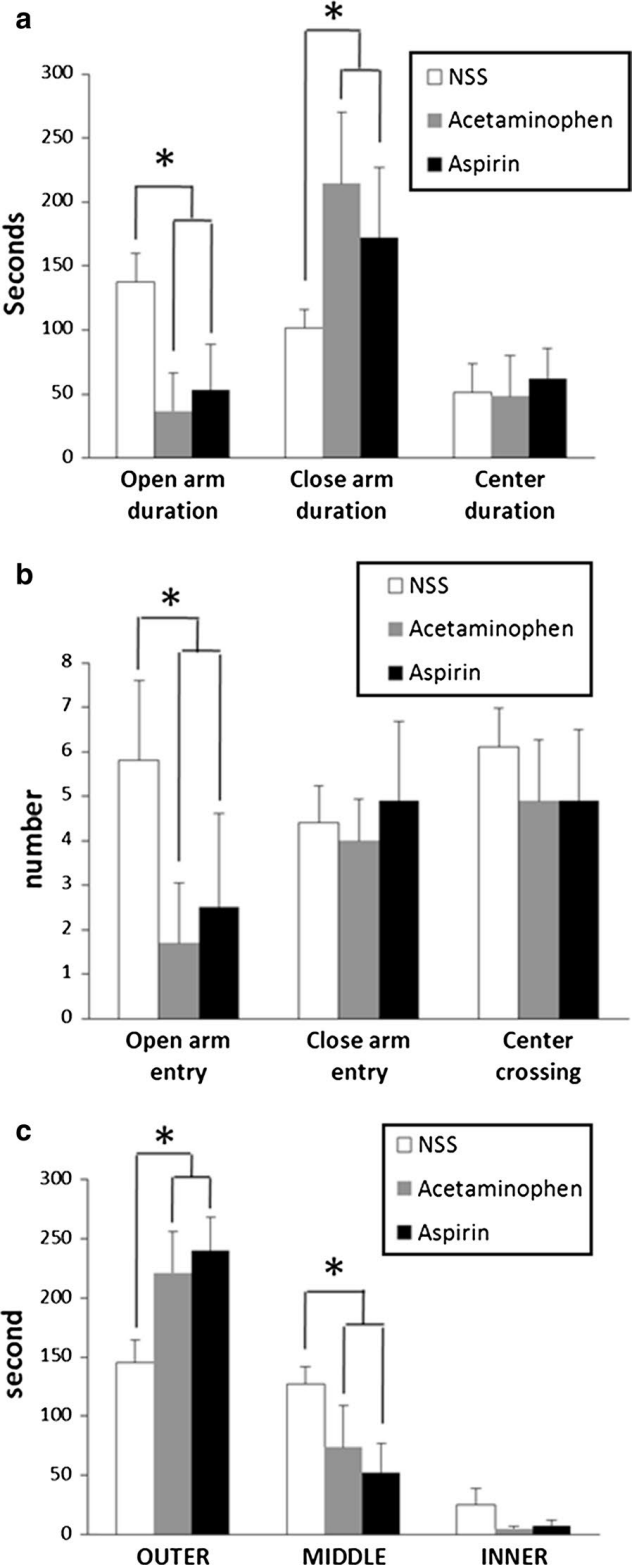
The effect of chronic treatment of rats with analgesics on the duration (**a**) and number of open-arm entries (**b**) on an elevated plus maze, and open field test results (**c**) (mean ± SD for 10 rats). *P < 0.05 compared with normal saline-treated control rats, one-way ANOVA followed by a Dunnett test

Chronic treatment with analgesics increased anxiety-like behavior in the elevated plus maze without affecting the locomotor function seen in the open field test. In the elevated plus maze test, the number of entries into open arms and time spent in the open arms were lower in aspirin- or acetaminophen-treated rats than those for saline-treated control rats, whilst the time spent in the closed arm was increased after treatment with either analgesic. Aspirin-treated rats spent 53 ± 36.1 s in the open arms, acetaminophen-treated rats spent 37 ± 29.5 s, and saline-treated rats spent 137 ± 22.6 s (*P* < 0.001; *F*_2,27_ 32.8; Fig. 1a; Additional file 1: Table S1). There was no significant difference in any variable between aspirin-treated or acetaminophen-treated rats. No significant difference was detected in the amount of central crossing by rats from any of the three groups. Aspirin-treated rats spent 62 ± 23.8 s in central area, acetaminophen-treated rats spent 49 ± 31.0 s, and saline-treated rats spent 52 ± 22.1 s (*P* = 0.48; *F*_2,27_ 0.75; Fig. 1b).

Consistent with the elevated plus maze results, the open field test also indicated an increase in anxiety-like behavior by rats chronically treated with an analgesic. Rats chronically treated with either aspirin or acetaminophen tended to stay on the periphery of the field without entering the center. The time spent in outer areas by rats chronically treated with either analgesic was significantly greater than time spent by rats treated with saline (240 ± 28.4 s for aspirin-treated rats, 221 ± 34.4 s for acetaminophen-treated rats, and 145 ± 19.0 s for saline-treated control rats; *P* < 0.001; *F*_2,27_ 32.02; Fig. 1c). The numbers of total crosses by rats in the three groups were comparable (132 ± 51.3 s for aspirin-treated rats, 131 ± 26.5 for acetaminophen-treated rats, and 133 ± 10.4 s for saline-treated rats, *P* = 0.997; *F*_2,27_ 0.003; Fig. 1c). There was no significant difference in the number of crosses by rats treated with either analgesic (Fig. 1c).

### Effect of chronic treatment with analgesics on electrical properties of neurons in the amygdala

**Fig. 2:**
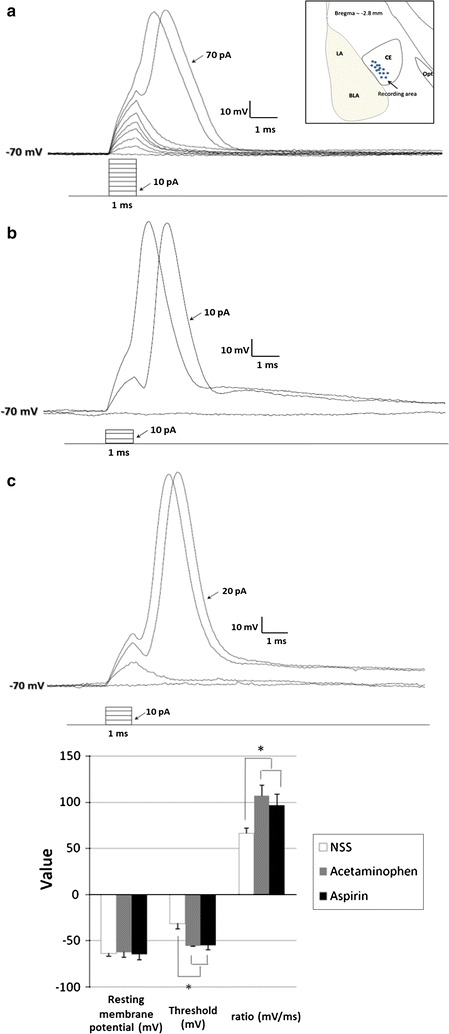
Representative traces of action potentials from neurons in the lateral central nucleus of the right amygdala (CeL) from rats chronically treated with the analgesics, and from saline-treated control rats. **a** Control, **b** acetaminophen, and **c** aspirin. The location of recorded neurons is shown in the *inset*. The *bar graph* compares the resting membrane potential, action-potential threshold, and ratio of rapid depolarization time and signal amplitude (mean ± SD of 10 animals). The threshold for action-potential generation in CeL neurons from rats chronically treated with aspirin or acetaminophen was more negative than that in control neurons from saline-treated rats. *P < 0.05, one-way ANOVA followed by a Dunnett test

A total of 30 neurons (10 neurons per group) were selected from the lateral subdivision of the central nucleus of the right amygdala (CeL). All selected neurons had to meet stringent criteria for health and stability. These healthy neurons had an input resistance between 180 and 220 MΩ and access resistance <16 MΩ. The holding potential of each neuron was –70 mV. Incrementally increasing current was injected as brief (1 ms) current pulses in a ladder of 10 pA steps until an action potential was generated. The threshold was considered as the first step generating an action potential. Chronic treatment of rats with analgesics increased the excitability of the neurons in their CeL (Fig. 2). The thresholds for action potential generation in neurons from aspirin-treated (–54.6 ± 5.01 mV) and acetaminophen-treated (–55.2 ± 0.97 mV) rats were more negative than for neurons from saline-treated vehicle control rats (–31.5 ± 5.34 mV). *P* < 0.001; *F*_2,27_ 99.67. Neurons in the CeL from rats chronically treated with analgesics depolarized faster than those from saline-treated control rats. The ratio of signal amplitude and rapid depolarization time was greater in rats chronically treated with analgesics (97.2 ± 11.69 mV/ms for neurons from aspirin-treated rats, 107.5 ± 10.87 mV/ms for neurons from acetaminophen-treated rats, and 66.5 ± 5.54 mV/ms for neurons from saline-treated rats. *P* < 0.001; *F*_2,27_ 47.85). There was no significant difference in the resting membrane potential of the neurons from rats in the three groups. The resting membrane potential was –64.2 ± 6.44 mV for neurons from aspirin-treated rats, –62.0 ± 5.53 mV for neurons from acetaminophen-treated rats, and –64.0 ± 2.70 mV for neurons from saline-treated rats, (*P* = 0.58, *F*_2,27_ = 0.56).

### Effect of chronic treatment with analgesics on CSD generation

**Fig. 3:**
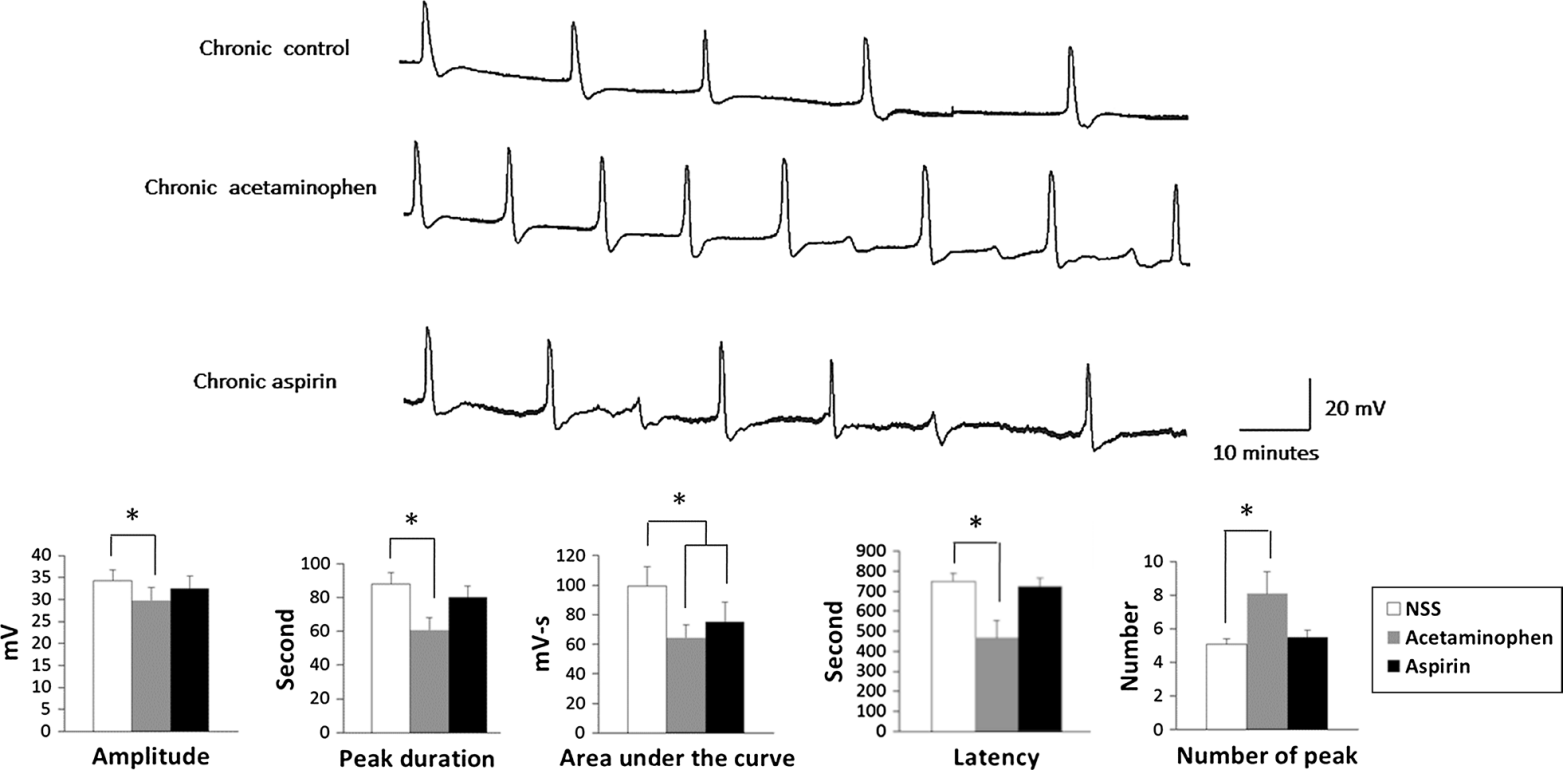
Representative cortical spreading depression DC potential response to KCl applied onto the dura mater of rats chronically treated with acetaminophen or aspirin, and saline-treated control rats. The *lower panel* compares variables including peak amplitude, duration, latency, and number, and area under the* curve*. *P < 0.05 compared with the CSD response in saline-treated control rats, one-way ANOVA followed by a Dunnett test

Chronic treatment with aspirin or acetaminophen altered the pattern of CSD generation (Fig. 3; Additional file 1: Table S2). This effect was more prominent in acetaminophen-treated rats (n = 10 per group). The CSD depolarization waves in rats chronically treated with acetaminophen were more frequent and had diminished duration, area-under-the curve, and interpeak latency. The presence of small, poorly developed CSD waves was observed in rats treated with either analgesic (Fig. 3). The average number of CSD waves developed in the first hour was 6 ± 0.4 in aspirin-treated rats, 8 ± 1.3 in acetaminophen-treated rats, and 5 ± 0.3 in saline-treated control rats (*P* < 0.001; *F*_2,27_ 39.43; Fig. 3). A similar CSD pattern was observed between acetaminophen- and aspirin-treated rats, but the area-under-curve was significantly smaller in acetaminophen-treated rats (Fig. 3).

### Effect of chronic treatment with analgesics on CSD-evoked Fos expression

**Fig. 4:**
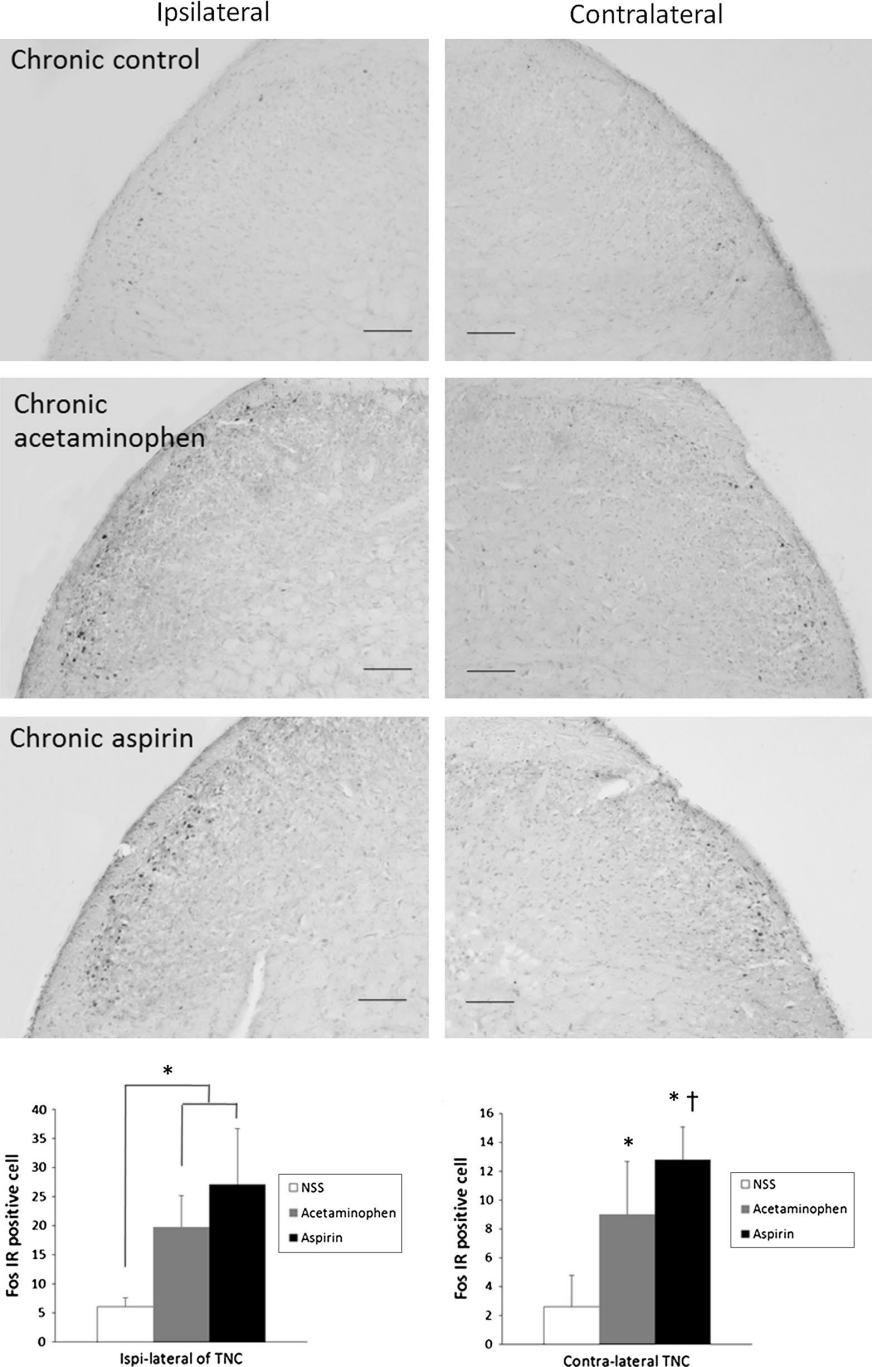
Cortical spreading depression-evoked Fos-immunoreactive neurons in the trigeminal nucleus caudalis of rats chronically treated with acetaminophen or aspirin, and normal saline-treated control rats (*scale bar* 100 μm). The *graph* presents a statistical analysis of Fos-immunoreactive neurons (mean ± SD of counts per slide from 10 rats). *P < 0.05 compared with saline-treated control rats, one-way ANOVA followed by a Dunnett test. ^†^ *P* < 0.05 between aspirin and acetaminophen treated rats, one-way ANOVA followed by a Dunnett test

Chronic treatment with analgesics significantly increased CSD-evoked Fos expression in both sides of the TNC with greater prominence on the side ipsilateral (right) to the CSD stimulus (Fig. 4; Additional file 1: Table S3). In the ipsilateral (right) TNC of aspirin-treated rats there were 27 ± 9.6 Fos-IR neurons per slide, 20 ± 5.4 neurons per slide in acetaminophen-treated rats, and 6 ± 2.2 neurons per slide in saline-treated control rats (*P* < 0.001; *F*_2,27_ 27.81). Only the contralateral (left) side of the TNC of aspirin-treated rats had significantly (*P* = 0.04) more Fos-IR cells than acetaminophen-treated rats.

**Fig. 5:**
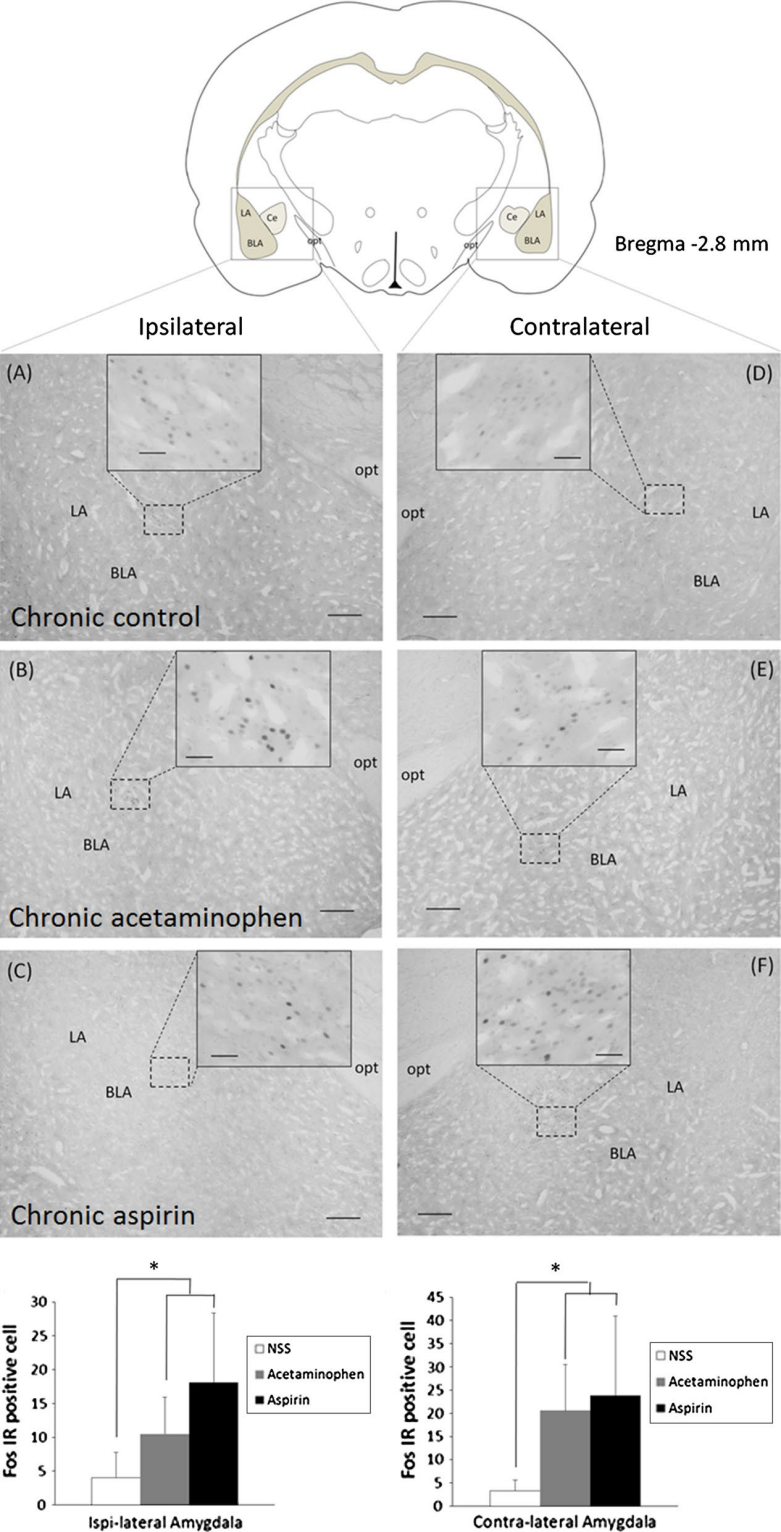
Cortical spreading depression-evoked Fos-immunoreactive neurons in the central nucleus of the amygdala from rats chronically treated with acetaminophen or aspirin, and from saline-treated control rats (*scale bar* 250 μm in each section and 50 μm in the *inset*). **a** Ipsilateral control, **b** ipsilateral chronic acetaminophen, **c** ipsilateral chronic aspirin, **d** contralateral control, **e** contralateral chronic acetaminophen and **f** contralateral chronic aspirin. The *graph* presents a statistical analysis of Fos-immunoreactive neurons (mean ± SD of counts per slide from 10 rats). *P < 0.05 compared with neurons in the amygdala of saline-treated control rats, one-way ANOVA followed by a Dunnett test

 Chronic treatment with analgesics increased Fos expression in both the ipsilateral (right) and contralateral (left) amygdala (Fig. 5; Additional file 1: Table S3). Fos-IR neurons were confined to the capsular region of the central amygdala. In the ipsilateral (right) amygdala there were 18 ± 10.2 Fos-IR neurons per slide in aspirin-treated rats, 11 ± 5.4 neurons per slide in acetaminophen-treated rats, and 4 ± 3.7 neurons per slide in saline-treated control rats, *P* < 0.001; *F*_2,27_ 10.04). Unlike the TNC, a greater degree of change was observed in the contralateral (left) amygdala. In the contralateral (left) amygdala there were 24 ± 17.2 Fos-IR neurons per slide in aspirin-treated rats, 21 ± 9.9 neurons per slide in acetaminophen-treated rats, and 3 ± 2.3 neurons per slide in saline-treated control rats (*P* < 0.001; *F*_2,27_ 8.65). There was no significant difference in the numbers of Fos-IR neurons between amygdala from aspirin- or acetaminophen-treated rats.

## Discussion

Chronic treatment with the analgesics induced significant changes in the system that controls emotion including anxiety. Animals receiving chronic treatment with the analgesics showed anxiety-like behavior and increased activity in the neurons of amygdala. Chronic treatment with analgesics also enhanced the development of CSD and facilitated the activity of neurons in the trigeminal nociceptive pathway and in the central nucleus of the amygdala.

Anxiety is common in patients with chronic headache associated with analgesic overuse. This association may be causally related, either chronic headache causing anxiety or the converse. In this paradigm, an index disorder causes or predisposes individuals to developing the comorbid disorder [16]. Therefore, this association must be unidirectional in nature [16]. However, epidemiological and clinical evidence suggests that the association between chronic headache and psychiatric disorders is bidirectional. Psychiatric disorders are found to be significant risk factors for development of MOH [16], and coexisting psychiatric disorders predict a worse outcome of MOH treatment [17, 18]. This bidirectional association implies that both MOH and anxiety are the result of chronic analgesic exposure. The presence of anxiety-like behavior in rats chronically treated with analgesics is consistent with this bidirectional association hypothesis.

In the present study, we demonstrated that neurons of the central amygdala nucleus of rats chronically treated with analgesics have a decreased action potential threshold. The amygdala plays an important role in fear and anxiety [19]. Each amygdala can be subdivided into three major nuclei, namely the lateral nucleus, basolateral complex, and central nucleus [19]. The lateral nucleus receives multimodal sensory information from the thalamus and cortex, and projects to the central amygdala via the basolateral complex. The central amygdala serves as a major output station. Neurons in the central nucleus send outputs to several brainstem and hypothalamic nuclei involved in the generation of conditioned fear responses [19]. Bilateral destruction of the central amygdala results in a decrease of fear-related behavior and decreased levels of corticotrophin-releasing factor in the cerebrospinal fluid and decreased levels of adrenocorticotropic hormone in the plasma [20]. Bilateral lesions of central amygdala also prevent visceral (bladder) hyperalgesia, which was induced by acute stress (foot shock) [21]. By contrast, increased activity of neurons in the central amygdala has been observed in models of chronic pain. For instance, neurons in the central amygdala of arthritic rats developed increased excitability compared with vehicle- or untreated controls [22]. A decreased action potential threshold of neurons in the central amygdala in rats chronically treated with analgesics as shown in the present study suggests that the activity of this nucleus is facilitated after prolonged exposure to analgesics. This facilitation may underlie the development of anxiety seen in patients with MOH.

We showed that in addition to evoking Fos expression seen as immunoreactivity in the TNC, CSD also evoked Fos expression in the capsular region of central amygdala. This region has been defined as the “nociceptive amygdala” because it relays nociceptive specific information from the spinal cord and brainstem via spino-(trigemino-)parabrachio-amygdaloid pathway [23]. Neurons in this region of the central amygdala may preferentially respond to noxious stimuli. Interestingly, the activity of neurons in this region can be modified by calcitonin gene-related peptide (CGRP) that has an important part in migraine pathogenesis [24]. Application of CGRP receptor antagonists inhibits the synaptic plasticity of this region in brain slices from arthritic rats. These effects on plasticity were accompanied by decrease of neuronal excitability and reduction of amplitude, but not frequency, of miniature excitatory postsynaptic currents compared with those obtained from normal controls [24]. The observation of CSD-evoked Fos expression in the central amygdala together with expression of Fos in the TNC implies the involvement of the capsular region of the central amygdala in trigeminal nociception. We note that application of KCl to the dura mater can also directly activate dural nociceptive fibers. Therefore, Fos can be expressed in the TNC as a result of either process.

We showed that chronic treatment with aspirin or acetaminophen had similar, but not identical, effects on excitability of neurons. Acetaminophen increased the excitability of cortical neurons more strongly than aspirin, while aspirin induced greater expression of Fos in the TNC and amygdala. That chronic treatment with aspirin increased the level of anxiety-like behavior without altering CSD suggests that there is no direct causal relationship between CSD and anxiety. These differences in the effects of aspirin are consistent with clinical observations that although several classes of medication can induce chronic headache, the features of MOH following different acute headache medications overuse are not exactly the same. A daily tension-type headache usually develops in patients overusing ergots and analgesics, while those overusing triptans were more likely to have frequent migraine-like headaches [25]. Unfortunately, to our knowledge there are no currently available data for the effect of different drugs in inducing psychiatric symptoms in patients with MOH.

## Conclusions

Chronic exposure to analgesics can increase the excitability of neurons in the central nucleus of the amygdala, which may underlie the development of anxiety or depression seen in patients with MOH. Our findings suggest that psychiatric symptoms coexisting with MOH should be considered as an epiphenomenon related to medication overuse and are not a direct consequence of chronic pain.

## Additional file

**Additional file 1:** **Table S1**. The effect of analgesic exposure on CSD anxiety behaviors. **Table S2.** The effect of analgesic exposure on CSD generation. **Table S3**. Number of Fos-immunoreactive cells evoked by CSD.

## Abbreviations

5-HT: 5-hydroxytrptamine
CGRP: calcitonin gene-related peptide
CSD: cortical spreading depression
DC: direct current
IR: immunoreactivity
MOH: medication-overuse headache
PBS: phosphate-buffered saline
TNC: trigeminal nucleus caudalis
