# Supplementary material for: Neural hyperactivity in the amygdala induced by chronic treatment of rats with analgesics may elucidate the mechanisms underlying psychiatric comorbidities associated with medication-overuse headache
Source: BMC Neurosci. 2017 Jan 3;18:1. doi: 10.1186/s12868-016-0326-z (PMC5209916; doi:10.1186/s12868-016-0326-z)
Supplement: Supplementary file 1 — Additional file 1: Table S1. The effect of analgesic exposure on CSD anxiety behaviors. Table S2. The effect of analgesic exposure on CSD generation. Table S3. Number of Fos-immunoreactive cells evoked by CSD. [file 12868_2016_326_MOESM1_ESM.docx]

**Supplementary table 1. The effect of analgesic exposure on CSD anxiety behaviors**

| **variable** | **Control** | **Acetaminophen** | **Aspirin** | **P value** |
| --- | --- | --- | --- | --- |
| **Elevated Plus Maze** |  |  |  |  |
| Open arm entry | 5.8+1.8 | 1.7+1.4 | 2.5+2.1 | < 0.001 |
| Open arm duration | 137.6+22.6 | 36.7+29.5 | 53.0+36.1 | < 0.001 |
| Closed arm entry | 1.4+0.8 | 4.0+0.9 | 4.9+1.8 | 0.298 |
| Closed arm duration | 101.5+14.2 | 214.2+55.8 | 172.2+55.1 | < 0.001 |
| Central crossing | 6.1+0.9 | 4.9+1.4 | 4.9+1.6 | 0.080 |
| Central duration | 51.5+22.1 | 48.5+31.0 | 62.1+23.8 | 0.481 |
| **Open field test** |  |  |  |  |
| Outer | 145.1+19.02 | 221.1+34.4 | 239.7+28.4 | < 0.001 |
| Middle | 127.2+14.3 | 73.8+34.9 | 52.5+24.2 | < 0.001 |
| Inner | 25.4+13.4 | 5.2+1.7 | 7.6+5.2 | <0.001 |
| Crossing | 132.5+10.4 | 131.3+26.5 | 131.8+51.3 | 0.997 |

**Supplementary table 2. The effect of analgesic exposure on CSD generation**

| **variable** | **Control** | **Acetaminophen** | **Aspirin** | **P value** |
| --- | --- | --- | --- | --- |
| Amplitude (mV) | 34.21+2.51 | 29.75+3.05 | 32.50+2.87 | 0.006 |
| Duration (second) | 87.85+7.14 | 60.88+7.39 | 80.19+6.76 | <0.001 |
| Area-under-curve (mV-s) | 99.37+9.21 | 64.40+8.84 | 75.36+13.11 | <0.001 |
| Latency (second) | 748.09+41.12 | 467.74+85.09 | 725.81+41.46 | <0.001 |
| Number of wave | 5.0+0.3 | 8.2+1.3 | 5.8+0.4 | < 0.001 |

**Supplementary table 3. Number of Fos-immunoreactive cells evoked by CSD**

| **Group** | **Control** | **Acetaminophen** | **Aspirin** | **P value** |
| --- | --- | --- | --- | --- |
| TNC- ipsilateral | 6.1+2.2 | 19.8+5.4 | 27.1+9.6 | <0.001 |
| TNC - contralateral | 2.6+2.2 | 9.0+3.6 | 12.8+2.2 | < 0.001 |
| Amygdala- ipsilateral | 4.0+3.7 | 10.5+5.4 | 18.1+10.2 | <0.001 |
| Amygdala- contralateral | 3.3+5.3 | 20.6+9.9 | 23.8+17.2 | <0.001 |
